# Effect of cervical suspensory traction in the treatment of severe cervical kyphotic deformity

**DOI:** 10.3389/fsurg.2022.1090199

**Published:** 2023-01-06

**Authors:** Pan Shengfa, Chen Hongyu, Sun Yu, Zhang Fengshan, Zhang Li, Chen Xin, Diao Yinze, Zhao Yanbin, Zhou Feifei

**Affiliations:** ^1^ Department of Orthopaedics, Peking University Third Hospital, Beijing, China; ^2^ Engineering Research Center of Bone and Joint Precision Medicine, Beijing, China; ^3^ Beijing Key Laboratory of Spinal Disease Research, Beijing, China

**Keywords:** severe cervical kyphosis, traction, correction, cervical kyphotic deformity, effect

## Abstract

**Objective:**

This study aimed to investigate a new noninvasive traction method on the treatment of severe cervical kyphotic deformity.

**Methods:**

The clinical data of patients with severe cervical kyphosis (Cobb > 40°) treated in Peking University Third Hospital from March 2004 to March 2020 were retrospectively summarized. 46 cases were enrolled, comprising 27 males and 19 females. Fifteen patients underwent skull traction, and 31 patients underwent suspensory traction. Among them, seven used combined traction after one week of suspensory traction. Bedside lateral radiographs were taken every two or three days during traction. The cervical kyphosis angle was measured on lateral radiographs in and extended position at each point in time. The correction rate and evaluated Japanese Orthopedic Association (JOA) scoring for the function of the spinal cord were also measured. The data before and after the operation were compared with paired sample t-test or Wilcoxon signed-rank test.

**Results:**

No neurological deterioration occurred during the skull traction and the cervical suspensory traction. There were 12 patients with normal neurological function, and the JOA score of the other 34 patients improved from 11.5 ± 2.8 to 15.4 ± 1.8 at the end of follow up (*P* < 0.05). The average kyphotic Cobb angle was 66.1° ± 25.2, 28.7° ± 20.1 and 17.4° ± 25.7 pre-traction, pre-operative, and at the final follow-up, respectively (*P* < 0.05). The average correction rate of skull traction and suspensory traction was 34.2% and 60.6% respectively. Among these, the correction rate of patients with simple suspensory traction was 69.3%. For patients with a correction rate of less than 40% by suspensory traction, combined traction was continued, and the correction rates after suspensory traction and combined traction were 30.7% and 67.1% respectively.

**Conclusions:**

Pre-correction by cervical suspensory traction can achieve good results for severe cervical kyphotic deformity, with no wound and an easy process. Combined traction is effective for supplemental traction after suspensory traction.

## Introduction

Cervical kyphosis can cause neck pain, myelopathy, and radiculopathy, and can affect swallowing and breathing (1). It can be congenital or can occur as a result of laminectomy for spinal cord tumor, cervical tubercular spondylitis, vertebral tumor, idiopathically, or as a post-trauma deformity (2). In all these situations, there is the potential for progressive deformity and the development of myelopathy because of compression or impingement of the spinal cord. It is generally accepted that surgical correction is warranted in cases of progression of kyphosis or neurological decline (3). The correction of cervical kyphosis has proven challenging because of the close proximity of important structures such as the vertebral artery, trachea, and esophagus (4), especially in severe cases which were defined as a Cobb angle >40°.

The strategy for correcting cervical kyphosis has been controversial (5). Scholars generally recognize the importance of intraoperative traction because it can facilitate intubation and the initial surgical exposure (6), especially in severe cervical kyphosis. However, traction pre-correction is not a consensus in pre-operative preparation. In fact, pre-operative traction can slowly reduce the degree of cervical kyphosis (7), thus avoiding nervous system damage caused by the sudden correction in the operation. In addition, a smaller degree of kyphosis before surgery and a smaller degree of correction during surgery can significantly reduce the risk of mechanical complications such as implant extraction (8).

Some researchers have recommended pre-operative halo-gravity traction or skull bone traction to pre-correct kyphosis before the operative treatment, thereby reducing the difficulty and complication rate of the operation (9). However most patients cannot tolerate the long time of staying in bed for bone traction. In view of the shortcomings of traditional skull traction, such as its invasiveness, poor patient tolerance, and many traction-related complications, we designed a different traction mode, namely cervical suspensory traction. In this retrospective longitudinal study, we investigated the radiographic and clinical outcomes as well as the safety of the traditional skull traction and noninvasive suspensory traction in the treatment of severe cervical kyphotic deformity.

## Materials and methods

### Study design

We retrospectively reviewed 46 patients with severe cervical kyphosis (defined as Cobb angle of kyphotic region >40°) underwent surgical treatment at our hospital between March 2004 and March 2020. A single surgeon performed all the operations. Demographics and baseline clinical variables were collected from the electronic medical records.

### Traction procedure

The enrolled patients were divided into three groups according to different pre-operative traction modes (Table 1). The specific traction procedures are shown below.

**Figure 1:**
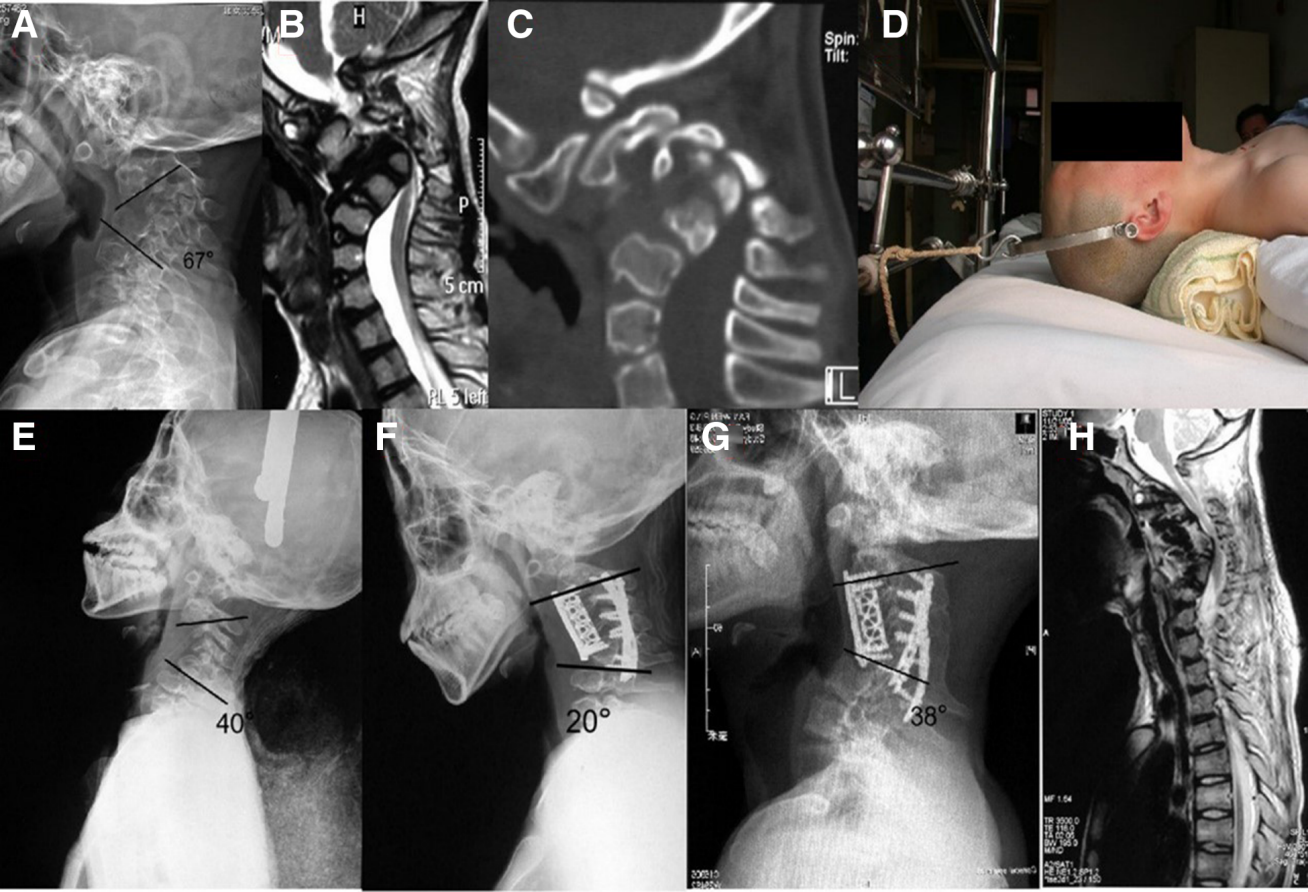
Case 1. This 15-year-old male complained of tetraplegia caused by osteochondrodysplasic cervical kyphotic deformity. (**A**) Pre-operative x-ray revealed a C2–7 Cobb angle of 67° before traction. (**B**) A para-sagittal computed tomography (CT) reconstruction showed no facet joint fusion. (**C**) Sagittal magnetic resonance imaging (MRI) showed severe compression of spinal cord at C3–5 level. (**D**) Skull bone traction. (**E**) Two weeks after skull bone traction, the Cobb angle had reduced to 40°; the correction rate was 40.3%. (**F**) Post-operative x-ray showed the Cobb angle reduced to 20°. (**G**) x-ray at 4 years showed progression of kyphosis at C6–7 level. (**H**) MRI at 4 years showed incomplete decompression of cervical spinal cord with Japanese Orthopedic Association scale (mJOA) score improved from 11 to 13 post-operatively.

**Figure 2:**
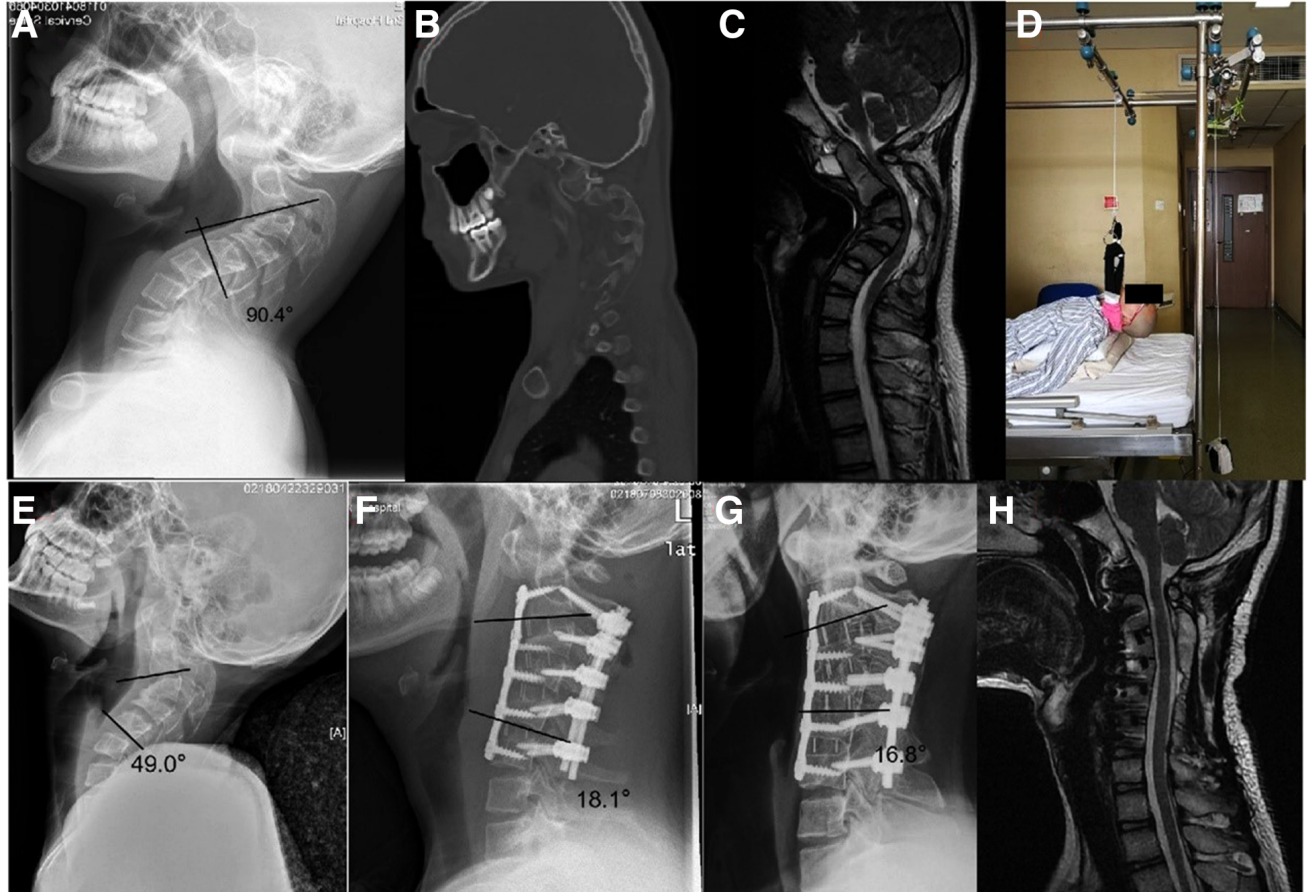
Case 2. This 14-year-old male complained of neck pain and weakness of the hands due to myelopathy caused by idiopathic cervical kyphotic deformity. (**A**) Pre-operative x-ray revealed a C2–5 Cobb angle of 90.4° before traction. (**B**) A para-sagittal computed tomography (CT) reconstruction showed C3–4 facet joint fusion. (**C**) Sagittal magnetic resonance imaging (MRI) showed severe compression of spinal cord at C2–4 level. (**D**) One week suspensory traction. (**E**) After one week of suspensory traction, the Cobb angle had reduced to 49°; the correction rate was 45.8%. (**F**) Post-operative x-ray showed the Cobb angle reduced to 18.1°. (**G**) x-ray at 1 year showed no loss of correction. (**H**) MRI showed completed decompression of cervical spinal cord with the modified mJOA score improved from 14 to 17 post-operatively.

**Figure 3:**
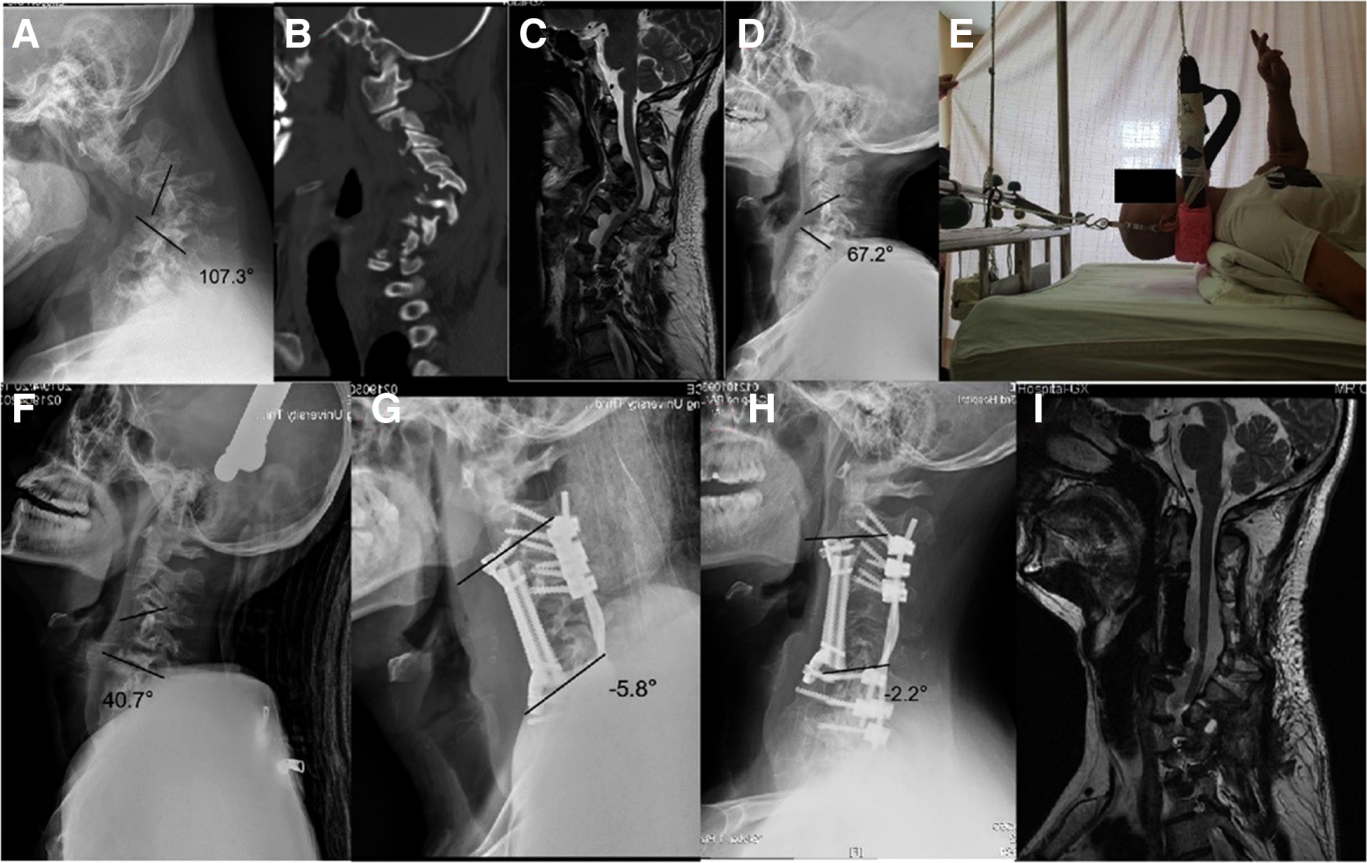
Case 3.  A 44-year-old patient with severe NF-1 cervical kyphosis had tetraplegia after an unexpected fall. Pre-operative mJOA score was 10. (**A**) Pre-operative x-ray revealed a C3–7 Cobb angle of 107.3° before traction. (**B**) A para-sagittal computed tomography (CT) reconstruction showed no facet joint fusion. (**C**) Sagittal magnetic resonance imaging (MRI) showed severe compression of spinal cord at C3–6 level. (**D**) After one week suspensory traction, the Cobb angle had reduced to 67.2° with a correction rate of 37.4%. (**E**) 12 days combined traction. (**F**) After 12 days combined traction, the Cobb angle had improved to 40.7° with a correction rate of 62.1%. (**G**) Post-operative x-ray showed the Cobb angle reduced to −5.8°. (**H**) x-ray at 2 years showed no loss of correction. (**I**) MRI showed completed decompression of cervical spinal cord with mJOA score improved from 10 to 14.5 post-operatively.

1. Gardener-Wells skull bone traction: Conventional axial continuous skull traction was applied to the patient (Figure 1). The weight of bone traction starts at 2–3 kg and gradually increases thereafter. The maximum weight is not more than one-fifth of the patient's body weight. In the process of traction, observe the sensation and movement of the patient's limbs and instruct the patient to report to the doctor immediately if they have numbness and weakness of the limbs. The patients undergoing skull traction were defined as group A.
2. Cervical suspensory traction: The patient was positioned supine on the orthopedic bed. A thoracic blanket roll was placed under the shoulders. The 10 cm-wide strap, instead of the Gardener-Wells tongs, provided a vertical traction at mid neck (Apex of kyphotic cervical spine) (Figure 2). The weight applied using two pulleys started from 1 kg and was gradually increased to 2–3 kg at the end of first day, then by 1–2 kg per day until it reached about 20% of the body weight or the tolerance threshold was reached. The minimun traction time was 8 h per day, and the patient could eat and sleep normally at other times. Skin was inspected regularly. Neurological examinations were performed three times per day, and if the patient had any complaint during the traction, the traction weight was reduced temporarily with the traction maintained. Lateral cervical radiograph was obtained every two or three days to evaluate the effect of the traction. The patients undergoing only cervical suspensory traction were defined as group B.
3. Combined traction: After one week suspensory traction, if the reduction rate was less than 40%, another skull traction was added (Figure 3), starting from 3 kg and gradually increasing over three days until about 20% of the patient's body weight was reached. Then, we decreased the weight of suspensory traction, where the maximum total traction weight should be less than 30% of patient's body weight, and the duration of combined traction continued up to 1 to 2 weeks. All the patients needed skull bone traction during the operation. The patients undergoing suspensory traction and combined traction were defined as group C.

**Table 1:** Group according to traction mode.

Group	Traction procedure	Patient number
A	Gardener-Wells skull bone traction	15
B	Cervical suspensory traction	24
C	Cervical suspensory traction + Combined traction	7

During daytime traction, patients were told to do push-and-pull trachea exercises; in the case of children, a family member was able to help them with this.

### Clinical data

Of the 46 patients, 37 experienced neck pain. 31 patients had cervical myelopathy, 10 had radicular symptoms in addition to myelopathy. Clinical outcomes were assessed using the mJOA score (10), visual analog scale (VAS), and Neck Disability Index (NDI).

### Radiographic evaluation

**Figure 4:**
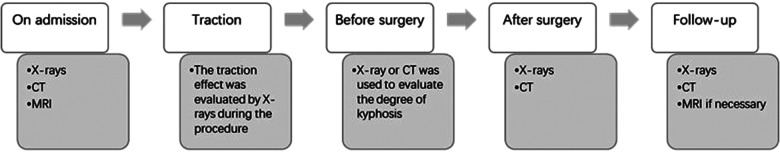
Radiographic evaluation of the patients. Radiographic evaluations were applied at five time points: admission, during traction, before surgery, after surgery, and at follow-up.


Radiographic outcomes were obtained at pre-operative, post-operative, and follow-up assessments (Figure 4). These outcomes were evaluated by cervical x-rays and thin-cut computed tomography (CT) scans at all three assessments. Para-sagittal section of CT images were used to estimate facet joint ankylosing. The cervical curvature of operation region was measured using the 2-line Cobb method (11). Magnetic resonance imaging (MRI) of the cervical spine was also obtained in all patients for further investigation of the intraspinal contents and compressive pathological feature. Correction rate (CR)is defined as the ratio of the difference between the Cobb angle before and after correction in relation to the Cobb angle before correction

$$\left(\mathrm{CR}=\frac{\begin{array}{c} \mathrm{Cobb}\;\mathrm{angle}\;\;\mathrm{before}\;\\ \mathrm{correction} \end{array}-\begin{array}{c} \mathrm{Cobb}\;\;\mathrm{angle}\;\;\mathrm{before}\;\\ \mathrm{correction} \end{array}}{\mathrm{Cobb}\;\;\mathrm{angle}\;\;\mathrm{before}\;\;\mathrm{correction}}\right)$$

### Operative technique

All patients underwent intraoperative skull bone traction. Motor-evoked potential and somatosensory-evoked potential monitoring were used during the operation of 28 (60.9%) patients.

Thirty patients underwent anterior multiple discectomy, corpectomy, or a hybrid technique of corpectomy combined with discectomy. Three patients underwent posterior column osteotomy (PCO) before correction, including to loosen facet joints, Smith-Peterson osteotomy (SPO), and Ponte osteotomy. Thirteen patients underwent circumferential surgery. Among these, three patients received 540° decompression and fusion.

### Statistical analysis

Data were managed and analyzed using SPSS Version 19.0 [International Business Machines (IBM), Armonk, NY]. Data were presented as mean ± SD. Paired sample t-test and Wilcoxon signed-rank test were used to test for significant differences in normally and non-normally distributed data, respectively. A *P*-value <0.05 was considered statistically significant.

## Results

The retrospective study comprised 46 patients: 27 males and 19 females. The mean age at the time of surgery was 18.7 ± 11.1 years (range, 5–69 years). The mean duration of follow-up was 25.8 ± 19.1 months (range, 12–84 months) (Table 2). Two patients were lost to follow-up at one year post-operation.

**Table 2:** Patient demographic data *N *= 46.

Characteristic	Value
Gender (M/F)		27/19
Age (years), mean ± SD (range)		18.7 ± 11.1 (5–69)
Body weight, mean ± SD (range)		48.2 ± 10.5 (15–70)
Pathology, *n* (%)	Neurofibromatosis type 1	12 (26.1%)
	Idiopathic	10 (21.7%)
	Vertebral congenital fusion	9 (19.6%)
	Iatrogenic	8 (17.4%)
	Osteochondrodysplasia	4 (8.7%)
	Posttraumatic	2 (4.3%)
	Cerebral palsy	1 (2.2%)
Follow-up period (months) mean ± SD (range)		25.8 ± 19.1 (12–84)

SD, standard deviation.

### Traction consequence

Fifteen patients received 9.8 ± 3.0 (6–15) days of skull traction. Thirty-one patients received 7.4 ± 1.1 (6–10) days of cervical suspensory traction; the traction weight was 6.9 ± 1.4 kg (3.5–9 kg). Among them, seven patients were treated with combined traction for another 1–2 weeks. Nine patients had pin-site pain during skull traction. No neurological deterioration occurred during the cervical suspensory traction and combined traction. Three patients had mild dizziness, and four patients had nausea at the early time of suspensory traction, and there were some patients who suffered mild headache during combined traction. These uncomfortable symptoms could be alleviated by reducing traction weight or time. No pin-related complications (e.g., pin loosening, pin-site infection) were reported.

### Clinical outcomes

Intraoperative spinal cord monitoring did not show any abnormal findings during the surgery. No spinal cord injury occurred. At post-operative and final follow-up, mJOA, VAS of neck pain, and NDI had improved significantly compared with the pre-operative scores (*P* < 0.05; Table 3). The scores of the last follow-up further improved significantly compared with post-operative assessment.

**Table 3:** mJOA, VAS of neck pain, and NDI scores.

Parameter	Pre-traction	Post-traction	Post-operation	Last follow-up
mJOA (*n* = 34)	11.5 ± 2.8	11.9 ± 2.4	13.5 ± 2.3*	15.4 ± 1.8**
VAS of neck pain (*n* = 46)	3.4 ± 1.3	NA	0.9 ± 1.0*	1.0 ± 0.8**
NDI (*n* = 46)	17.5 ± 6.3	NA	10.4 ± 4.9*	8.4 ± 3.6**

mJOA, modified Japanese Orthopedic Association scale; VAS, Visual Analog Scale; NDI, Neck Disability Index.

* *P* < 0.05 compared with Pre-traction.

** *P* < 0.05 compared with post-operation.

### Radiographic parameters

Thirty-one (67.4%) patients had no facet joint fusion on pre-operative para-sagittal CT reconstruction, seven (15.2%) patients showed one segmental fusion, and five (10.9%) patients showed two segmental fusions.

After Gardener-Wells skull bone traction, the Cobb angle decreased from pre-traction (pre-trac) 63.9 ± 19.7 degrees to pre-operation (pre-op) 42.8 ± 17.7 degrees. The correction rate (CR) was 34.2%. Post-operative Cobb angle was 20.5 ± 12.0 degrees. The post-operative correction rate was 68.6%. The final Cobb angle was 21.6 ± 13.1 degrees in last follow up.

After cervical suspensory traction, the Cobb angle decreased from pre-traction (pre-trac) 67.2 ± 27.7 degrees to post-suspensory-traction (post-sus-trac) 29.9 ± 21.1 degrees. *P* < 0.01. The correction rate (CR) was 60.6%. Then, seven patients were treated with combined traction. The correction rate before the operation was 68.8%. Post-operative Cobb angle was 11.6 ± 18.5 degrees. The post-operative correction rate was 85.6%. The final Cobb angle was 15.4 ± 30.0 degrees in last follow up (typical case Figures 2, 3). There was no correction loss in final follow-up (Table 4).

**Table 4:** The change of Cobb angle in operative region (°).

	N	Pre-trac	Post- sus-trac	CR (%)	Pre-op	CR (%)	Post-op	CR (%)	Final follow-up	CR (%)
Total	46	66.1 ± 25.2	—	—	28.7 ± 20.1*	57.5 ± 23.1	14.6 ± 17.0*	80.1 ± 17.1	17.4 ± 25.7	77.3 ± 22.2
Group A	15	63.9 ± 19.7	—	—	42.8 ± 17.7	34.2 ± 10.9	20.5 ± 12.0	68.6 ± 11.7	21.6 ± 13.1	67.0 ± 13.7
Group B	24	58.2 ± 14.4	18.3 ± 12.3	69.3 ± 11.5	18.3 ± 12.4	69.3 ± 11.5	7.0 ± 8.0	88.4 ± 14.1	7.6 ± 8.8	88.0 ± 14.0
Group C	7	98.3 ± 40.0*	69.5 ± 31.6*	30.7 ± 7.1*	34.4 ± 27.2*	67.1 ± 16.0	28.2 ± 32.5*	76.1 ± 22.1	42.2 ± 55.7*	62.6 ± 39.1**
Group B + C	31	67.2 ± 27.7	29.9 ± 21.1	60.6 ± 23.9	21.9 ± 17.7	68.8 ± 18.5	11.8 ± 18.5	85.6 ± 16.7	15.4 ± 30.0	82.3 ± 23.9

* *P* < 0.01compared with Group B.

** *P* < 0.05 compared with Group B.

Twenty-four patients from Group B received single suspensory traction. The correction rate post traction was 69.3%. Seven patients from Group C had more serious kyphosis than the patients from Group B (*P* < 0.01). After one week of suspensory traction, Group C achieved a correction rate of 30.7 ± 7.1% compared with Group B 69.3 ± 11.5% (*P* < 0.01). After continued combined traction, Group C further improved up to the operation and achieved almost the same correction rate (67.1 ± 16.6% compared with Group B 69.3 ± 11.5%.). There was no statistical significance between the post-operative correction rate of the two groups; Group C had a lower correction rate than Group B at the final follow-up (*P* < 0.05).

### Complications

During Gardener-Wells skull bone traction, nine patients (60%) experienced pin-site pain. During suspensory traction, three patients (9.7%) experienced minor dizziness, four (12.9%) minor emesis. There was no occurrence of pin tract infection or pin loosening in combined traction patients. No neurological deteriorations were reported during traction.

Surgery related complications were noted in seven (22.6%) of 31 patients in Group B and C: one with transient quadriplegia, one with dura tear who needed lumbar drain, and one with superficial infection who needed debridement. The latter patient unfortunately experienced Atlanto-axial instability and needed occipitocervical-thoracic fusion after one year. Two had minor dysphagia at the last follow-up.

## Discussion

The surgical correction methods of cervical kyphosis can be roughly categorized into anterior approach, posterior approach, and combination, which depend on patient clinical status and imaging characteristics (2). Patients with severe cervical kyphosis have contracture of the surrounding soft tissue, increased spinal intramedullary pressure, and spinal nerve compression (12), leading to increased surgical risk and complications.

Mummaneni et al. (13) retrospectively analyzed 30 cases of cervical kyphosis treated by combined anterior-posterior surgery and found that the correction and neurological function improvement were satisfactory, but the incidence of complications, such as cervical plate dislodgement, pseudarthrosis, and dysphonia, was as high as 33%. Lau et al. (14) reported that the mechanical complication rate after posterior osteotomies for correction of moderate to severe adult cervical deformity was 28.9%. A review noted that the intraoperative complication rate of cervical deformity correction was 39.9% (15).

Scholars generally recognize the importance of intraoperative traction because it can facilitate intubation and initial surgical exposure, especially in severe cervical kyphosis. However, in pre-operative preparation, traction pre-correction is not a consensus. In fact, pre-operative traction can slowly reduce the degree of cervical kyphosis, thus avoiding the nervous system damage caused by sudden orthopedic surgery. In addition, a smaller degree of kyphosis before surgery and a smaller degree of correction during surgery can significantly reduce the risk of mechanical complications such as implant extraction. Due to the level of difficulty and high complication rate of surgical correction of severe kyphosis, some scholars began to use traction to reduce the degree of cervical kyphosis before surgery and found that the clinical effect was satisfactory (16). The purpose of pre-operative traction is to achieve slow correction of deformity. It effectively avoids abrupt distension of nerves to minimize the risk of neurologic compromise (8). Pre-operative traction can also partially correct cervical deformity, thus providing an easier surgery. Helenius IJ et al. (7) found that pre-operative traction did not significantly affect the occurrence of post-operative complications. However, the degree of cervical kyphosis in the pre-operative traction group was higher than that in the non-traction group.

Dynamic flexion-extension radiographs have been used to determine whether the cervical spine was fixed in the past, thus affecting treatment including traction and surgery. However, this method is not reliable. Para-sagittal CT reconstruction was performed at our institution to assess the presence of fusion in the patient's lateral mass. Of the 43 patients in this study, most (72.1%) had no fusion, and the others had only one- or two-level fusion. Cervical kyphosis can be defined as flexible rather than fixed in all patients in this study and can therefore be corrected pre-operatively by suspensory traction.

In this study, it was found that cervical suspensory traction could significantly reduce the degree of cervical kyphosis before surgery. The CR was 60.6%, which is a satisfying result compared to that of skull traction or halo-gravity traction reported in previous studies (Table 5). Satisfactory correction only by suspensory traction occurred in 77.4% of patients. In addition, the average traction time was 7.4 days, which was significantly lower than that of traditional skull traction in some previous studies (17,18, 22).

**Table 5:** Clinical results of pre-operative traction in different studies.

	Patient Number	Angles Before Traction, mean ± SD^a^	Traction	Number of traction days, mean (range)	Angles After Traction, mean ± SD^a^	Correction (%)	Complications
Sink 2001 (17)	22	97°	Halo-gravity traction	91 (42–196)	72°	25.8	7/22 Pin loosening, pin-site infections, or numbness
Kawabata 2013 (18)	3	97.6°	Halo-gravity traction	30	52°	46.7	1/3 numbness in the upper extremities worsened
Zeng 2015 (19)	20	30.8° ± 10.5°	Halo-gravity traction	4.8 (3–7)	2.9 °±3.9°	90.6	8/20 ASIA classification improved one grade
Helenius IJ 2016 (7)	9/22	70°	Halo-gravity traction	25 (6–65)	46°	34	0/9 new nerve deficits
Shen X 2019 (20)	33	71.7° ± 18.5°	Skull traction	6.6 (5–9)	32.4° ± 11.6°	54.8	—
Verhofste 2019 (21)	28	91° ± 20.7°	Halo-gravity traction	25 (13–29)	56° ± 17.6°	38	9/28 superficial pin-site infection, transient paresthesia, or preexisting neurological deficit
Yankey 2021 (22)	37	Cobb2–7: 43.12° ± 20.08°	Halo-gravity traction	28	Cobb2–7: 25.94° ± 16.37°	39.8	0/37 neurological deficits
Group A	15	63.9 ± 19.7	Skull bone traction	9.8 (6–15)	42.8 ± 17.7	34.2	9/15 pin-site pain
Group B	24	58.2° ± 14.4°	Suspensory traction	7.4 (6–10)	18.3° ± 12.3°	69.3	7/24 minor dizziness or minor emesis
Group C	7	98.3° ± 40.0°	Suspensory traction and skull traction	17.8 (15–22)	After suspensory traction: 69.5° ± 31.6° After skull traction: 34.4° ± 27.2°	After suspensory traction: 30.7 After skull traction: 67.1	0/7 pin tract infection or pin loosening

^a^ Unless otherwise specified, all angles represent kyphotic angles.

In order to ensure that the patients who were not satisfied with the correction effect after suspensory traction (CR<40%) could get adequate pre-correction before surgery, skull traction was added. The cervical spine received both a forward corrective force and an upward axial force. To avoid the patient being unable to tolerate combined traction, the force of suspensory traction was reduced while adding skull traction. The study found that where it was difficult to pre-correct by suspensory methods, patients could achieve the same CR as other patients after the addition of combined traction.

Dysphagia is a common post-operative complication of cervical deformity surgery (23). Therefore, patients in our institution were told to do push-and-pull trachea exercises when they were in traction. This method can stretch the soft tissue of the anterior neck contracture and enhance the patient's tolerance to corrective surgery.

When some scholars use halo-gravity traction to correct cervical kyphosis, the target weight was 30%–50% of body weight (21). However, the traction force we used was lower. On the one hand, many of the patients with severe cervical kyphosis are children (74% of the patients were <18 years in this study) and cannot tolerate big traction force. On the other hand, patients with cervical kyphosis caused by neurofibromatosis type 1 can experience osteopenia or osteoporosis (24), and strong traction can easily cause bone destruction.

Due to the need for pins inserted into the skull in traditional axial traction, pin loosening, pin-site infection, and brain abscess have been reported in previous studies. In contrast, suspensory traction, as a noninvasive procedure, does not cause complications related to pins and is more accepted by patients. Suspensory traction exerts a posterior rotational force on the cervical spine, thus lengthening the anterior contracted soft tissue. The posterior structures such as the facet joints and the muscles of the back of the neck are not subjected to much traction, and the corresponding neck discomfort will not occur.

### Limitations

Due to the small number of patients included in this study, subgroup analysis of the etiology of cervical kyphosis and flexibility could not be performed. The design of single-center retrospective analysis has potential selection bias and recall bias.

## Conclusion

Noninvasive suspensory traction can effectively reduce the cervical kyphosis angle of patients with severe cervical kyphosis. Combined traction can be used as a supplementary pre-operative corrective measure for poor corrective results after suspensory traction.

## Data Availability

The raw data supporting the conclusions of this article will be made available by the authors, without undue reservation.
